# Reliable B Cell Epitope Predictions: Impacts of Method Development and Improved Benchmarking

**DOI:** 10.1371/journal.pcbi.1002829

**Published:** 2012-12-27

**Authors:** Jens Vindahl Kringelum, Claus Lundegaard, Ole Lund, Morten Nielsen

**Affiliations:** 1 Center for Biological Sequence Analysis, Technical University of Denmark, Lyngby, Denmark; 2 Instituto de Investigaciones Biotecnológicas, Universidad de San Martín, San Martín, Buenos Aires, Argentina; La Jolla Institute for Allergy and Immunology, United States of America

## Abstract

The interaction between antibodies and antigens is one of the most important immune system mechanisms for clearing infectious organisms from the host. Antibodies bind to antigens at sites referred to as B-cell epitopes. Identification of the exact location of B-cell epitopes is essential in several biomedical applications such as; rational vaccine design, development of disease diagnostics and immunotherapeutics. However, experimental mapping of epitopes is resource intensive making *in silico* methods an appealing complementary approach. To date, the reported performance of methods for *in silico* mapping of B-cell epitopes has been moderate. Several issues regarding the evaluation data sets may however have led to the performance values being underestimated: Rarely, all potential epitopes have been mapped on an antigen, and antibodies are generally raised against the antigen in a given biological context not against the antigen monomer. Improper dealing with these aspects leads to many artificial false positive predictions and hence to incorrect low performance values. To demonstrate the impact of proper benchmark definitions, we here present an updated version of the *DiscoTope* method incorporating a novel spatial neighborhood definition and half-sphere exposure as surface measure. Compared to other state-of-the-art prediction methods, *Discotope-2.0* displayed improved performance both in cross-validation and in independent evaluations. Using *DiscoTope-2.0*, we assessed the impact on performance when using proper benchmark definitions. For 13 proteins in the training data set where sufficient biological information was available to make a proper benchmark redefinition, the average AUC performance was improved from 0.791 to 0.824. Similarly, the average AUC performance on an independent evaluation data set improved from 0.712 to 0.727. Our results thus demonstrate that given proper benchmark definitions, B-cell epitope prediction methods achieve highly significant predictive performances suggesting these tools to be a powerful asset in rational epitope discovery. The updated version of *DiscoTope* is available at www.cbs.dtu.dk/services/DiscoTope-2.0.

## Author Summary

The human immune system has an incredible ability to fight pathogens (bacterial, fungal and viral infections). One of the most important immune system events involved in clearing infectious organisms is the interaction between the antibodies and antigens (molecules such as proteins from the pathogenic organism). Antibodies bind to antigens at sites known as B-cell epitopes. Hence, identification of areas on the surface antigens capable of binding to antibodies (also known as B-cell epitopes) may aid the development of various immune related applications (e.g. vaccines and immunotherapeutic). However, experimental identification of B-cell epitopes is a resource intensive task, thereby making computer-aided methods an appealing complementary approach. Previously reported performances of methods for B cell epitope predictive have been moderate. Here, we present an updated version of the B-cell epitope prediction method; *DiscoTope*, that on the basis of a protein structure and epitope propensity scores predicts residues likely to be involved in B-cell epitopes. We demonstrate that the low performances to some extent can be explained by poorly defined benchmarks, and that inclusion of additional biological information greatly enhances the predictive performance. This suggests that, given proper benchmark definitions, state-of-the-art B cell epitope prediction methods perform significantly better than generally assumed.

## Introduction

The interaction between antibodies and antigens has been the center of attention for multiple disciplines within immunological research and applications [1] [2], and a dozen of methods for computational mapping of antibody binding on the antigen surface (B-cell epitopes) have been developed in the later years. However, the performance of these methods has in general been moderate [3] [4].

Methods for predicting B-cell epitopes can in general be divided into two groups based on the level of information needed to do the prediction; methods utilizing information derived only from the protein sequence and methods using information from protein 3-dimentional structures. Traditionally, sequence based methods are build from calculations of hydrophilicity, flexibility, Beta-turns and surface accessibility [5] [6] [7] [8], and in recent years methods utilizing amino acid composition and amino acid cooperativeness have shown promising results [9] [10] [11]. While these methods perform reasonable when predicting epitopes composed of a continuous stretch of amino acid (linear epitopes), they fail to predict epitopes consisting of amino acids segments, distantly separated in the protein sequence and brought together by the conformational folding of the polypeptide chain (conformational epitopes).

Inclusion of structural information, to some extent, overcomes the shortcoming of sequence-based methods, as amino acid distant in sequence but close in space can be identified. Andersen and coworkers [12] investigated the performance of the Parker scale [6] and epitope amino acid composition as well as measures derived from the protein 3-dimentional structure for prediction of conformational epitopes and concluded that introduction of structural data significantly outperformed sequence based methods. The method developed, *DiscoTope*, acts by probing the carbon backbone of the protein structure under study with a 10 Å sphere, summing the propensity score of residues in the sphere and subtracting the neighbor count (number of amino acid residues within the sphere). Other methods define the structural neighborhood as the nearest surface exposed residues [13] or a patch on the surface of the protein [14] [15]. The introduction of structural data furthermore expands the number of physical-chemical and biological attributes that can be calculated and used for prediction [13] [16], as exemplified by the work of Rubinstein and coworkers [15]. In their work, Rubinstein et al. [15] calculated 45 attributes from the 3-dimentional structures of known epitopes and applied them for prediction. Interestingly, only a fraction of attributes (21/45) that previously had been proved to significantly distinguish epitope from non-epitope areas, proved to be important for prediction. Similarly, the EPSVR method developed by Liang and coworkers [13] implements 6 propensity scores in a support vector regression algorithm, of which three have been proved to be associated with antigenicity [17], and the remaining three with surface exposure. However, performance of the two methods, and other methods utilizing a vast number of features, still only achieve predictive performance values comparable to much simpler models employing two or three attributes [4] [12] [18] [19] [20]. In general, structural based methods are most successful when implementing features like amino acid composition [12] [15] [18], epitope amino acid cooperatively [15], secondary structure [13] [15] in combination with one or more surface measure e.g. RSA [21], neighbor count [12], half-sphere neighbor count [22], and protrusion index [23].

While structural information significantly improve predictions of B-cell epitopes, the use of protein structures introduce several major problems: First of all, even though the number of resolved antigen-antibody structures is increasing, data for building structure-based models are still scarce. Secondly, very few antigens have been extensively studied in order to map the exhaustive set of epitope residues. The existence of un-characterized epitopes makes it difficult to accurately evaluate the performance of prediction models, as even a perfect prediction will classify experimentally undetected epitopes as false positives. Furthermore, biologically relevant proteins are often parts of larger complexes, which behave as one unit in the biological environment that they are part of. However, structural information on the entire “biological unit“ is often not available, hence leading to a lack of information essential to correctly predict B-cell epitopes.

Here, we present an improved version of the structural based prediction method, *DiscoTope*, updated using a redefinition of the spatial neighborhood used to sum propensity scores and half-sphere exposure as a surface measure. Using this update method, we illustrate when and why predictions may fail and show that failed predictions, to some extent, can be explained by a poorly defined benchmark setup or an incomplete definition of the biologic unit responsible for the given antibody response.

## Results

The *DiscoTope* method [12] is driven by a combination of: 1) statistical difference in amino acid composition between epitope and non-epitope residues, calculated as log-odds ratios [24], 2) a definition of the spatial neighborhood for integrating log-odds ratios in a residue proximity and 3) a surface measure. As neither the definition of spatial neighborhood nor surface measures are trivial tasks, one aim of the presented work was to investigate the ability of a new scoring function for defining a spatial neighborhood and different surface measures to improve the accuracy for B-cell epitope prediction. Next, given such improved predictive performance, we aimed to demonstrate that changing the benchmark setup to include for each antigen information from multiple epitopes and the “biological unit” used to raise the antibody response significantly enhance the reported prediction power.

### Defining the spatial neighborhood: Predictions by log-odds ratios

**Figure 1:**
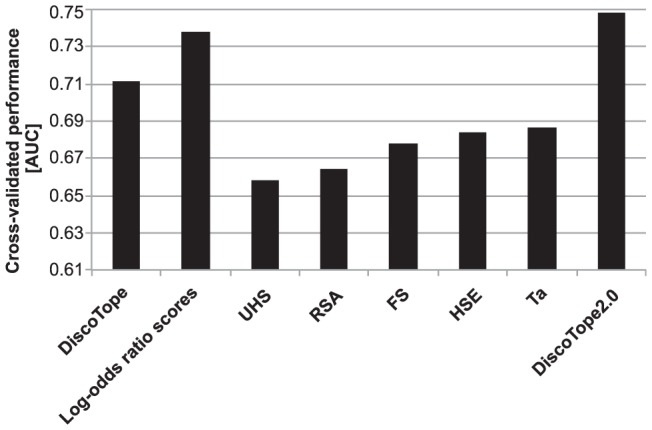
Cross-validated performance. Performances of different methods for predicting B-cell epitopes evaluated on the DiscoTope dataset. From left to right: The original *DiscoTope* method, the uncombined log-odds ratio scores as described in text, the surface measures; UHS, RSA, FS HSE and Ta (see text) and the *DiscoTope2.0* method as described in text. Performance of the original *DiscoTope* method was obtained from [12].

Several methods for predicting B-cell epitopes have successfully utilized the deviation in epitope and non-epitope amino acid composition [12] [15] [13] [10]. Here, epitope amino acid composition was calculated as the logarithm of the ratio between amino acid frequencies in epitope and non-epitopes, as described in Andersen et al. [12]. A novel scoring function, integrating amino acid log-odds ratios in the spatial proximity of a residue was used to calculate the combined log-odds ratio scores used for prediction. The function was inspired by the work of Andersen et al. [12] and Sweredoski and Baldi [18] and defines the neighborhood around each residue as a sum of neighboring log-odds ratios weighted by a function that decreased concurrently with distance. In difference to the function proposed by Sweredoski and Baldi [18], which uses 5 distance thresholds to stepwise decrease the weight on log-odds ratios, the function proposed here is defined by only two parameters: a sequential smoothing window *w* and a distance scale *k*_ps_ (for details see Material and Methods). The parameters were estimated by a 2-dimentional-grid search applied to the DiscoTope dataset described in [12], with optimal values *w* = 1±0 (i.e no smoothing) and *k_ps_* = 21.6±0.90 Å, respectively, where the values given are the mean and standard deviation from the 5 fold cross-validated training procedure. The optimal parameters were hence found to be stable between each data set in the cross-validation. Predictions of B-cell epitope by log-odds scores using this proximity sum function had a performance of AUC 0.738 (Figure 1).

### Predictions by surface measures

5 different surface measures calculated from the protein structure, were tested for their ability to discriminate epitope from non-epitope residues (see Materials and Methods and Table S3 for details). As illustrated in Figure 1, all measures had comparable predictive performance and no method significantly outperforming the others (p>0.11 in all cases).

### Combining surface and log-odds ratio scores

A weighted sum of proximity summed log-odds ratios and a surface measure was used to give an overall prediction score (for details see Materials and Methods). The best performance was achieved when combining log-odds ratio scores with neighbor count in upper half spheres (UHS), which had an average AUC of 0.748 on the DiscoTope dataset using the cross-validated benchmark procedure. This method outperforms the original *DiscoTope* method (0.711. *p = 0.0022*) and also all the uncombined methods (*p<0.028*). As the method is driven by main principles introduced in the original *DiscoTope* method, we name this method *DiscoTope-2.0*.

Surprisingly, the only two surface measures that significantly improved performance in combination with log-odds ratio scores were UHS and RSA, which individually had the lowest predictive power. However, the FS, Ta and HSE scores are significantly stronger correlated with the log-odds scores than the UHS and RSA scores (*p<10^−6^*, Pearson correlation coefficients of 0.37–0.39 for UHS, RSA and 0.51–0.55 and for FS, Ta, HSE, respectively). These results hence suggest that the UHS and RSA scores contain more complementary information to the log-odds scores compared to the FS, HSE and Ta scores, explaining why these surface measures are optimal in the combined model.

The gain in predictive performance between the *DiscoTope-2.0* model (combining surface measures and proximity summed log-odds score) and the proximity summed log-odds scores alone is relatively small (see Figure 1). This could suggest that the signal from the surface exposure to some degree is embedded in the log-odds scores, as also suggested from the correlation analysis above. The log-odds scores are calculated from the ratio of amino acids frequencies found in epitopic versus non-epitopic residues. As B cell epitopes by nature are most often exposed, the log-odds will contain an implicit bias towards commonly exposed amino acids. To investigate the effect of this bias, we recalculated the log-odds ratios excluding residues with a relative surface accessibility (RSA) below a threshold of 0.01, 0.05 and 0.10 respectively and retrained all parameters. Note, the set of epitopic residues have an average RSA value of 0.30. In this setup, the set of non-epitopic residues is hence altered to include only exposed residues (at different thresholds), hence lowering the preferential bias towards exposure in the log-odds scores. The predictive performance of log-odds scores alone decreased concurrently with an increase in surface exposure threshold (AUC 0.731, 0.704 and 0.656 for threshold 0.01, 0.05 and 0.10 respectively), and more weight was put on the surface measure scores when combining log-odds and surface measure scores (for details see Figure S1 and S2). The loss of prediction power by the recalculated log-odds scores could not be restored in combination with any of the 5 surface measures and the combined method did in all cases perform worse than the *DiscoTope-2.0* method using the original log-odds scores (data not shown). It is hence clear that the high performance of the log-odds scores to a very high degree can be contributed to the inherent signal discriminating between surface and non-surface amino acid preferences, and not to a signal discriminating epitopic from non-epitopic surface residues.

### Impacts of proper definition of benchmark data

**Figure 2:**
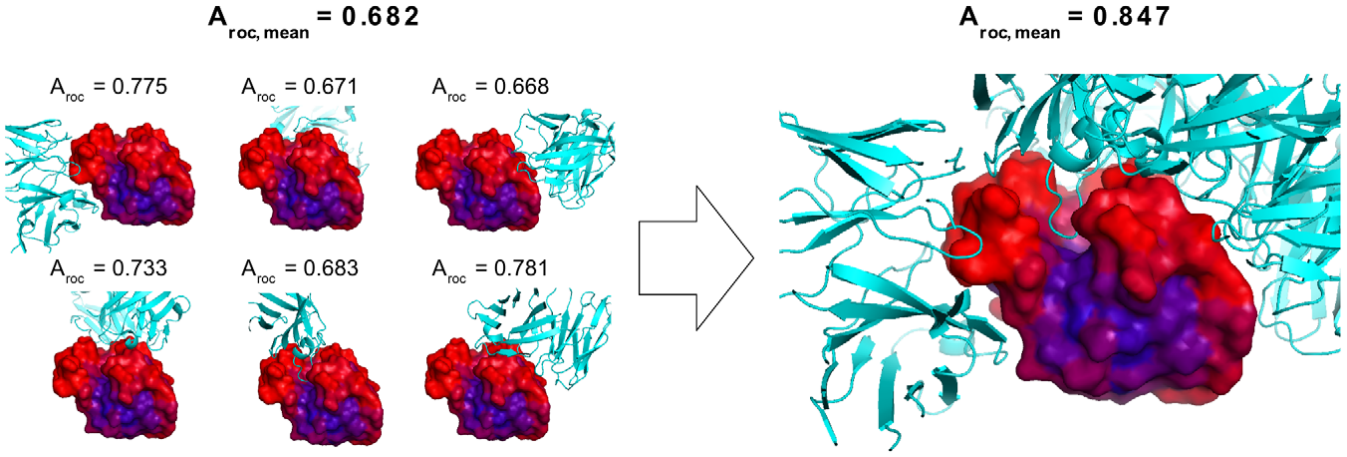
Illustration of benchmark redefinition on Lysozyme. 6 unique discontinuous epitopes have been identified for lysozyme. Including this comprehensive information on multiple epitopes for Lysozyme, the reported performance is increased. Predictions are illustrated as a heatmap on the protein surface where Red = high prediction score, Blue = low prediction score.

**Figure 3:**
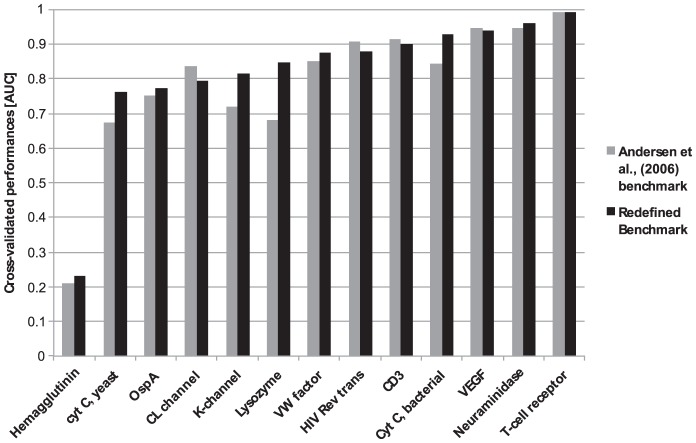
Effect of benchmark redefinition and inclusion of biological units in prediction accuracy for the subset of 13 affected homology groups (see text). Refer to Table S1 for complete definition of protein names.

A critical aspect of evaluation of a prediction model is the quality and consistency of the benchmark data set. In particular, incomplete annotations of benchmark data lead to artificially low estimates of the predictive performance due to positive predictions incorrectly being labeled as false positive. Having defined a high performing B cell epitope predictor, we can access the impacts of such incomplete benchmark definitions on the benchmark performance. The cross-validation benchmark setup used in this work for model development, as originally defined by Andersen and coworkers [12], suffers from several aspects of incomplete annotations. In the benchmark, each of the 75 antigen-antibody complexes in the DiscoTope dataset is treated as single entities ignoring the fact that the same antigen might contain several epitopes. Since each antigen-antibody complex is handled as a single entity, only the single epitopic region defined in the given complex is annotated as positive, ignoring other known epitopic regions defined in other antibody complexes with the same antigen. As earlier realized by Ponomarenko and Bourne [3] and Liang et al., [13], this annotation scheme is not optimal, and to evaluate how it impacts the predictive performance, AUC scores for antigens possessing more than one epitope (Table S1) were recalculated leaving out residues annotated as epitopes in other antigen∶antibody complexes included in the benchmark as previously described [25]. The effect was most dramatic illustrated by lysozyme that has 29 antigen-antibody complexes in the data set. Here the AUC score increased from 0.682 to 0.847 (Figure 2) when taking into account multiple definitions of epitopes. The AUC score for 5 of 6 affected proteins gained in performance, with an average increase of 0.039 (Figure 3). Furthermore, the number of non-similar epitopes mapped onto each antigen correlated significantly to the performance of DiscoTope-2.0 (Spearman's rank correlation coefficient of 0.33, p<0.01, exact permutation test). See Materials and Methods for definition of non-similar epitopes.

**Figure 4:**
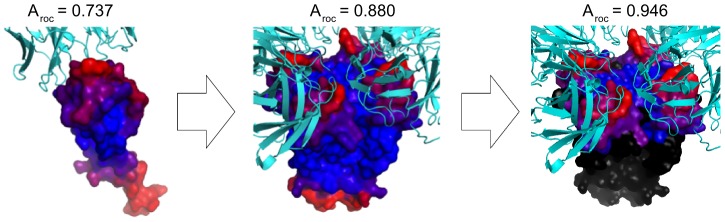
Enhance prediction accuracy by inclusion of structural data of the biological unit. Illustration of prediction for KvAP potassium channel. Left: using only one antigen chain, middle: using the biological tetramer, right: Excluding membrane and cytoplasmic residues. Predictions are illustrated as a heatmap on the protein surface where Red = high prediction score, Blue = low prediction score. Note, that the stated performances are for the PDB entry 1K4C and not the complete potassium homology group.

Another aspect of the benchmark definition that potentially has a large impact on the predictive performance is the data defining the neighborhood environment for each residue used to calculate the prediction score. Proteins are often parts of larger complexes, which behave as one biological unit. In most cases, antibodies are raised against the entire “biological unit”, and not only the part of the unit comprising the epitope. In the DiscoTope dataset described by Andersen et al., [12] only the chain interacting with the antibody is used to define the structural environment of the residues in the antigen. However, this might results in some residues being considered as highly exposed and predicted as epitopes, when they in reality are involved in complex formation with another chain and not accessible for the antibody. To investigate the impact on the predictive performance by including the biological unit rather than the single antigen chain, the performance for the subset of antigen complexes were recalculated where additional structural information on the biological unit was available in the PDB file using the biological unit as input. The 10 affected proteins had on average an increase in AUC of 0.020, with the KvAP potassium channel and cytochrome c proteins showing the largest increase (Figure 3). Figure 4 illustrates the change in prediction for the KvAP potassium channel. Using only the antigen∶antibody chains as input the performance of *DiscoTope-2.0* is 0.737. When including the whole biological unit, the value is increased to 0.880, and excluding residues categorized as cytoplasmic or trans-membrane (UniProt release 2012_01, www.uniprot.org), and thus not accessible for antibody binding the performance value is further increased to 0.946. The average performance of the 13 proteins (homology groups) affected by benchmark redefinition increased from an AUC of 0.791 to 0.824 (*p<0.035*) and the average performance of the entire *DiscoTope* dataset increased from an AUC of 0.748 to 0.765. Performances on each antigen in the *DiscoTope* dataset are presented in Table S1.

### Comparison to the PEPITO, ElliPro, SEPPA, Epitopia, EPCES and EPSVR prediction methods

Besides assessing the performance of DiscoTope-2.0 on the 75 antigen structures included in the DiscoTope dataset, the performance was assessed on an independent evaluation dataset extracted from the IEDB-3D database. The dataset consists of 52 antigen structures with no sequence overlap to the DiscoTope dataset (see Materials and Methods). To avoid bias towards antigens represented by multiple structures, the 52 structures were clustered into 33 homology groups based on antigen sequence similarity. The epitopes and benchmark procedure were initially defined in the same manner as for the DiscoTope dataset, hence only the chains interacting with the antibody were included (no biological unit) and multiple epitopes for the same antigen were treated as single entities (multiple epitopes are not accounted for).

The average predictive AUC performance of *DiscoTope-2.0* on the evaluation benchmark dataset was 0.731, which is higher than that of the original DiscoTope method (0.705). The difference is however not significant (*p = 0.086*). The evaluation dataset was furthermore used to compare the performance of DiscoTope-2.0 to the PEPITO (also known as BEpro) [18], ElliPro [19], SEPPA [26], Epitopia [14], EPCES [27] and EPSVR [13] methods, which are other recently developed methods for predicting conformational B-cell epitopes based on protein 3-dimentional data. The average AUC performance of DiscoTope-2.0 was significantly higher than that of ElliPro (0.686, *p = 0.041*) and comparable to that of PEPITO (0.732, *p = 0.53*). Comparison to the SEPPA, Epitopia, EPCES and EPSVR prediction methods were performed on subsets of the evaluation dataset not sharing sequence similarity to data used for training of the methods (Blast E-value<0.01). On these reduced benchmark dataset DiscoTope-2.0 showed improved AUC performance compared to SEPPA (0.720 vs 0.711, *p* = 0.34, 34 structures used) and EPCES (0.733 vs 0.695 *p* = 0.15, 49 structures used) and significantly improved performance compared to Epitopia (0.727 vs 0.652 *p* = 0.033, 43 structures used) and EPSVR (0.746 vs 0.588 *p* = 0.006, 24 structures used). The AUC values for DiscoTope-2.0, DiscoTope-1.2, PEPITO, ElliPro, SEPPA, Epitopia, EPCES and EPSVR on the evaluation dataset are available in supplementary materials Table S4. Note, that for the evaluation data set, only max four antibody∶antigen structures were available for each antigen. For the training data set this number was as high as 29 (for lysozyme). As shown before, these low numbers of antibody∶antigen structures for the antigens in the evaluation data set inherently translate into incomplete annotations of the epitopes contained within each antigen, and hence to an improper benchmark definition, leading to low benchmark performances.

The AUC value gives the overall predictive performance of a method integrated over the entire range of specificities. Often another relevant performance measure is how many of a given set of high scoring predictions are actual positive (the predictive positive value, PPV) and how large a fraction of the actual positives that are included in this set of predictions (the sensitivity). Given that an average B cell epitope contains 15 residues (Table S1), we calculated the average PPV and sensitivity values from the subset of top 15 and top 30 highest scoring predictions from each antigen for the different methods. The results of this analysis are shown in Table 1 for DiscoTope-2.0, DiscoTope-1.2, PEPITO and ElliPro using the entire benchmark dataset and in table S5 for SEPPA, Epitopia, EPCES and EPSVR using the subset of the benchmark dataset not used for training the different methods. These results confirm the overall earlier findings and consistent performance gain of the *DiscoTope-2.0* method compared to the other methods included in the benchmark both in terms of PPV and sensitivity.

**Table 1:** Predictive positive value (PPV) and sensitivity for *DiscoTope-2.0*, *DiscoTope-1.2*, *PEPITO* and *ElliPro* on the evaluation data set.

# Residues		*DiscoTope-2.0*	*DiscoTope-1.2*	PEPITO	ElliPro
15	PPV	0.190	0.191	0.184	0.145
	Sens	0.176	0.164	0.157	0.145
30	PPV	0.156	0.154	0.162	0.138
	Sens	0.280	0.252	0.274	0.253

# Residues gives the number of highest scoring prediction included for each antigen, PPV gives the predictive positive value (true positives)/(predicted positives)), and Sens gives the sensitivity (true positives)/(actual positives)).

In the evaluation dataset, additional structural information about the “biological unit” and/or multiple epitopes could be detected for 8 of the 33 homology groups. Including this additional information about the “biological unit” for prediction and redefining the benchmark setup to accommodate multiple epitopes, as described above for the training dataset, led to an statistically significant improvement in the average AUC for the 8 homology groups from 0.712 to 0.727 AUC (*p = 0.021*). Likewise, were the PPV and sensitivity values using the top 30 highest scoring predictions for each antigen increased from 0.168 to 0.188 (PPV) and 0.316 to 0.348 (sensitivity), respectively.

The overall findings on the DiscoTope dataset in terms of performance gain when including the biological unit for prediction and redefining the benchmark to accommodate multiple epitopes were hence confirmed on the evaluation set.

We investigate to what degree similar performance improvements were observed for the methods PEPITO, ElliPro and SEPPA when considering the “biological unit” for prediction and redefining the benchmark setup to accommodate multiple epitopes. Here, we find that only the PEPITO method has a performance gain whereas the SEPPA (which treats multi-chain inputs as independent queries) performance is unaltered and ElliPro (which applies the global shape of the input structure to estimate residue protrusion) displays a drop in predictive performance (data not shown).

## Discussion

Here, we have presented an updated version of the *DiscoTope* method for predicting discontinuous B cell epitopes. The update includes a novel definition of the spatial neighborhood used to sum propensity scores and half-sphere exposure as a surface measure.

Using the benchmark data set from the original DiscoTope paper, we demonstrate that the updated method has a significantly increased predictive performance. Several approaches to define the epitope log-odds propensity scale were investigated with the purpose of defining a score that could differentiate between epitopic and non/epitopic surface residues. However, the scale with the optimal performance was the original *DiscoTope* definition defined from the amino acid frequency in epitope residues compared to the frequency in non-epitopic residues [12]. Likewise, were several surface measures investigated for their ability to predict epitope residues. Here, the upper half-sphere exposure method gave the highest performance when combined with the proximity summed log-odds score.

The cross-validated predictive performance of *DiscoTope-2.0* on the DicoTope dataset [12] is 0.748. While this value is significantly different from random, the performance remains far from perfect. Many reasons exist for this relative low predictive performance. Here we argue that one very important, and often overlooked, reason stems from the definition of the data set. The *DiscoTope* benchmark data set consists of antigen∶antibody complexes found in the protein databank. Each epitope is defined from the crystal structure as the residues from the antigen structure that are in contact with one or more residues in the antibody structure. All other residues are annotated as non-epitopes. This definition is clearly highly simplistic and will in most cases lead to incomplete annotations, since other areas of the antigen surface than the given epitope might also bind antibodies [13] [19]. Another critical aspect of the benchmark definition lies in the data defining the neighborhood environment for each residue used to calculate the prediction score. The *DiscoTope* method defines epitopic residues from a combination of surface exposure and the log-odds propensity scores. The calculation of surface exposure for a given residue depends critically on the structural unit included to make the calculation. In the DiscoTope data set only the chain interacting with the antibody is used to define structural environment of the residues in the antigen. However, proteins are often parts of larger complexes, which behave as one biological unit, and antibodies are often raised against this entire “biological unit”, and not only the part of the unit comprising the epitope. For a subset of the data included in the DiscoTope benchmark, we can to some degree deal with both of these aspects and make a more precise definition of the benchmark data including information about the biological unit and/or multiple known epitopes. In doing this, the predictive performance is increased to 0.824.

Using an independent data set, we compared the performance of the updated *DiscoTope* method to that of the PEPITO, ElliPro, SEPPA, Epitopia, EPCES and EPSVR prediction methods. Here we find that *DiscoTope* and PEPITO achieved the highest predictive performance. Their performance was significantly higher that the ElliPro, Epitopia and EPSVR methods but not statistically significant different from that of the SEPPA and EPCES methods. More importantly however, we could demonstrate using the independent evaluation data set that including information about the biological unit for prediction and redefining the benchmark to accommodate multiple epitopes also here led to an improved predictive performance of the *DiscoTope* method. The gain in predictive performance when redefining the benchmark is smaller on the evaluation data set compared to that found for the training data. One major reason for this is that the characterization of the antigens in the evaluation data set is more incomplete to the “older” antigens of the training data set. The maximal number of antibody∶antigen structures for each antigen was thus four for the evaluation data set, whereas as this number for the lysosome antigen in the training data set was as high as 29. This low number of antibody∶antigen complexes available for each protein in the evaluation data set naturally translates into an overall under-estimation of the predictive performance.

Performing the same benchmark redefinition for the PEPITO method led to similar improved predictive performance whereas the performance of the SEPPA was unaltered and the ElliPro performance dropped. This change in impact of the redefinition of the benchmark on the predictive performance reflects general properties of the different methods. Both *DiscoTope* and PEPITO use a local exposure measure calculated from the local structural environment of a given residue to predict the epitope score. Including information about the biological unit of the antigen alters the local structural environment of residues in contact with neighboring chains in the biological units and hence alters the prediction score for these residues only. For Ellipro, the situation is very different. ElliPro defines protrusion on a global scale by approximating the protein shape to an ellipsoid and assigning a residue protrusion index from the local deviation from the ellipsoid. Using such an approach, inclusion of the biological units will alter the ellipsoidal fit and hence the entire scoring scheme for all residues not only the once in contact with neighboring chains in the biological units. Likewise, does the SEPPA method treats multi-chain inputs as independent queries, and hence cannot benefit from this additional information.

**Figure 5:**
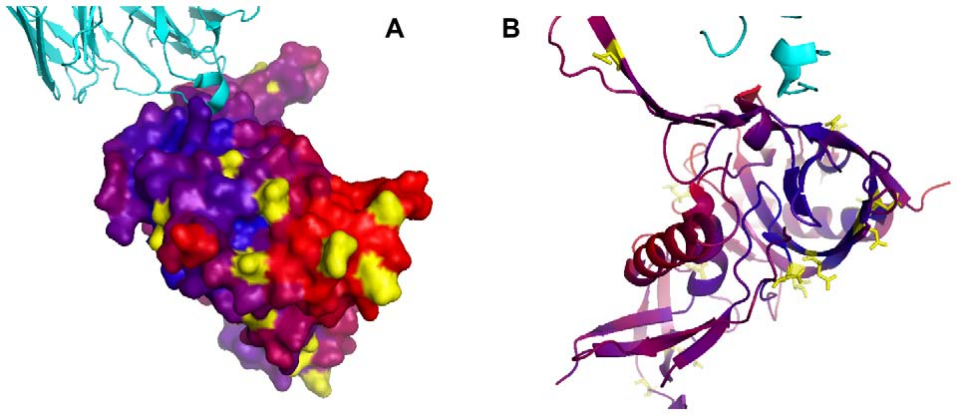
Predictions for Gp120 plotted on the protein structure including bound antibody. Each residue in the structure is colored from blue to red according to its *DiscoTope-2.0* score. Blue indicates low scores (predicted to be non-epitope residue) and red indicates high scores (predicted to be epitope residue). Yellow indicates possible glycosylation sites retrieved from UNIPROT accession number P04578 (www.uniprot.org). a) Gp120 surface representation and antibody cartoon representation. b) Gp120 and antibody cartoon representation. Note, the red alpha-1 helix, which is normally buried in the inner domain of Gp120 involved in Gp41∶Gp120 complex formation, is exposed in the crystal structure.

Examples where the *DiscoTope-2.0* method seems to fail completely are HIV-1 Gp120 core, and Influenza A Hemagglutinine (H3) (AUC<0.50). Both these proteins are glycoproteins residing on the virus envelope of influenza A and HIV respectively, and mediates the entrance of viral DNA into the host cell by binding to host cell surface proteins [28] [29]. Glycosylation patterns are not included in the resolved antigen∶antibody complexes, and the antibody chosen for complex formation must hence bind non-glycosylated sites of the antigen to be able to form the complex structure. This, since glycosylated sites would be shielded in the *in-vivo* environment where the antibody response was raised. In fact, mapping of potential glycosylation sites (as obtained from Uniprot accession number P04578 www.uniprot.org) onto the Gp120 structure revealed that the only non-glycosylated site predicted by *DiscoTope-2.0* to be antigenic, beside part of the antibody-binding site is the alpha-1 helix normally buried in the inner domain of Gp120 involved in Gp41∶Gp120 complex formation (Figure 5) [30]. Mapping of potential glycosylation sites (as obtained from Uniprot accession number P03437 www.uniprot.org) onto the Hemagglutine structures also excludes some of the sites predicted to be highly antigenic. The most prominent predicted antigenic site of Hemagglutinine is the active site residing in the ‘head’ region of the HA1 subunits. This site has been structurally recognized as an epitope in the PDB entries 3SM5, 2VIR 1KEN and 3LZF and antibodies binding to this epitope have a higher avidity for Hemagglutinin than the epitope included in the *DiscoTope* dataset. However, the structures were not included in the dataset as the structures fail the quality threshold of a maximum resolution of 3 Å (3SM5, 2VIR, 1KEN) or were submitted after to the PDB database after preparation of the *DiscoTope* dataset (3LZF).

The failed predictions for both Hemagglutinine and Gp120 can hence to some extent be explained by missing biological data and incomplete benchmark annotation and in both cases could the performance to a very high degree be recovered including information on glycosylation, the biological unit and other epitopic sites.

All antigen structures included in the benchmark study presented here are bound structures. This might to some extent impact our findings as the epitope area in the bound form of the antigen deviates slightly from the native form recognized by the antibody. However, the impact of such subtle structural changes will mainly impact methods relying on specific structural traits for prediction (like docking methods) where a significantly higher prediction performance in general is obtained on bound compared to unbounded structures. However, previously work (data not shown), suggests that for methods like PEPITO (BEpro), Epitopia and *Discotope*, that all rely on more coarse-grained structural features there is no different in performance between the bound and unbound antigen structure. We hence do not expect the issue to have major impact on results presented in this paper either. However, it should be noted that the methods EPCES and EPSVR were developed using primarily unbound antigen structures, and that the reported performances for these two methods hence might be underestimated.

In summary, we have described an improved version of *DiscoTope* for prediction of discontinuous B cell epitopes. Moreover, we have demonstrated that part of the reason for the relatively poor performance of state-of-the-art prediction methods for B cell epitopes can be attributed mostly to the quality of the benchmark dataset used. Taken together, we believe these observations underline firstly the importance of curated benchmark data sets of properly mapped structural B cell epitopes for the development and evaluation of methods for B cell epitope prediction, and secondly that, given such proper benchmark definitions, state-of-the-art prediction methods for B cell epitopes do have reliable and highly significant predictive performances.

The updated version of *DiscoTope* is available at www.cbs.dtu.dk/services/DiscoTope-2.0.

## Materials and Methods

### Data preparation

The *DiscoTope* dataset was used for method development as previously described [12]. In short; the dataset consist of 75 x-ray crystal structures of antigen-antibody complexes with a resolution <3 Å, divided into 25 homology-groups based on antigen sequence (Table S1). The 25 homology-groups were furthermore divided into five data sets used for training (4 sets) and evaluation (1 sets) in a cross-validation scheme. Epitopes were annotated as any residue in the antigen having atoms within a 4 Å distance to any atom in the antigen [12] [31]. Epitope annotation was downloaded from http://www.cbs.dtu.dk/suppl/immunology/DiscoTope and protein structures from the PDB database (www.pdb.org). The PDB files were further processed into 2 different files: 1) only containing the chain interacting with the antibody as original defined in the DiscoTope dataset and 2) PDB files containing additional structural information on the biological relevant unit (as described in the publication associated with the structure) if available (obtainable for PDB entry: 1XIW, 1TZH, 1CZ8, 1BJ1, 1K4D, 1K4C, 1KYO, 1EZV, 1NCA, 1NMC, 1A14, 1NCB, 1NCC, 1NCD, 1OTS, 1AR1, 1NFD, 2HMI, 1EO8, 1QFU). Details about this training data set, and the partition in to homology groups are listed in Table S1.

An independent evaluation dataset containing proteins not homologues to proteins in the DiscoTope dataset was constructed based on 584 PDB structures identified as antigen-antibody complexes in the IEDB database (http://www.immuneepitope.org/browse_by_3D.php?name=BCELL). Structure files (PDB files) were downloaded from the PDB database (www.pdb.org). Antibody heavy/light chains were automatically identified based on homology to two databases of antibody heavy and light chains respectively, from various organisms. Protein chains not identified as light or heavy chains were initially annotated as antigens. 132 PDB entries containing no protein antigen chain and 42 entries that did not have both light and heavy chains were discarded. 5 entries containing single-chained antibodies joining light and heavy chains were included. From the remaining set of 410 antigen-antibody complexes, 52 antigens were retrieved using the criteria: 1) Structure resolved by x-ray crystallization (405 entries), 2) Size of antigen chain >150 residues (136 entries) and 3) No sequence similarity overlap to antigens in the *DiscoTope* dataset (Blast E-values<0.01). The 52 PDB files were manual processed into files containing one copy of the biological unit (antibody and antigen) as described in the PDB entry. Epitope residues in the 52 antigens were annotated as described above for the *DiscoTope* dataset, and the antigens were clustered into 33 homology groups based on antigens sequence similarity. 2 entries were considered similar if any two antigen chains from the two entries had a blast value<0.01. Finally, the PDB files were processed into 2 different files containing: 1) The chains interacting with the antibody and 2) The biological relevant antigen unit, if available (obtainable for PDB entry: 3BSZ, 2ZJS, 2XTJ, 2FD6). Details of the evaluation dataset are given in Table S4 and the data are available at www.cbs.dtu.dk/suppl/immunology/DiscoTope-2.0.

### Derivation of epitope log-odds ratios

Log-odds ratios were calculated as previously described [12]. In brief: each antigen protein sequence was divided into a list of overlapping 9-mer peptides by sliding a window on the primary sequence. Next, the peptides were sorted into an epitope and a non-epitope group based on the annotation of the center residue. Amino acid weight matrixes for each group were then calculated by the method described in Nielsen et al., [24], using sequence clustering, sequence weighting and weight on pseudo counts of 200. Finally, log-odds ratios for each of the 20 amino acids were calculated from the central residue position (position 5) in the epitope weight matrix relative to the same position in the non-epitope matrix in means of half-bits. Surface corrected log-odds scores were calculated in a similar manner, by excluding peptides where the relative surface accessibility (RSA) for the central residue was below a predefined threshold. RSA thresholds of 0.01, 0.05 and 0.10 were used.

### Using log-odds ratios for epitope prediction – Definition of spatial neighborhood

For prediction of epitope residues, log-odds ratios were used in combination with a scoring function that sums the ratios of amino acids in the spatial neighborhood around each residue to give a log-odds ratio score for each residue in a given protein. Inspired by the work of Andersen et al., [12] and Sweredoski and Baldi [18], we defined a scoring function that decreases weight on log-odds ratios as a function of distance. The function used in the work by Sweredoski and Baldi [18] uses 5 distance thresholds to gradually decrease weight on log-odds ratios, which were set empirically to 8, 10, 12, 14, and 16 Å. Here, we designed a simpler function with a single distance threshold and furthermore included the smoothing window size *w*. This parameter was set based on optimization of sequence-based predictions (*w = 9*) by Andersen et al., [12] and adopted by Sweredoski and Baldi. The proximity sum (PS) function is defined below
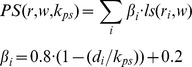
where *r* is the query residue for which the log-odds ratio score (PS) is computed, *r_i_* is any residue within *k_ps_* distance from *r*, *ls(r_i_,w)* is the log-odds ratio value of *r_i_*, sequentially averaged over a window of *w* residues and *d_i_* is the distance between *r* and *r_i_*. To ensure that log-odds ratios included in the neighborhood sphere influence the final score, the minimal weight was set to 0.2, which have been proved successfully in previously similar scoring functions [18]. A 2-dimentional grid search were applied to find the optimal set of parameters using the grids: *w = {1,3…11}*, *k_ps_ = {4,6…28 Å}*. Distance between two resides, were calculated as the distance between C_α_ atoms.

### Using surface measures for epitope prediction

5 different surface measures, calculated from the protein structure, were trained and tested for their ability to predict B-cell epitopes. These were variation of residue contact counts: Full sphere neighbor count (FS) [12], Upper half-sphere neighbor count (UHS) [22] and Half-sphere exposure as described in [22] (HSE) and previously used for B-cell prediction in [18]. A residue were classified as neighbor to the query residue if the C_α_ - C_α_ distance were below *k_sur_*. We furthermore tested the widely used relative surface accessibility (RSA) [21] and a hybrid between neighbor count and RSA (Ta) by defining neighbor residues as residues holding any atom within *T* distance of any atom in the query residue. Scoring functions and parameters are listed in Table S3. Neighbor count in upper and lower half-spheres were calculated using the structural bio-python module developed by T. Hamelryck [32], and surface accessibility calculated by DSSP using the standard 4 Å probe. The RSA were then obtained by dividing the surface accessibility with the maximum surface accessibility, calculated from the peptide GGXGG, where X is the amino acid in question. The optimal sphere radius *k_sur_*, for the FS, UHS, RSA, and HSE, and the distance threshold *T* for Ta were estimated by a grid search using the grids; *k_sur_ = {4,6…28 Å}* and *T = {4,6…28 Å}*.

### Combining log-odds and surface measure

The log-odds ratio scores were combined to each of the tested surface measures to give an overall prediction score. The scores were weighted according to the equation:
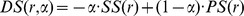
where *PS* and *SS* are the log-odds ratio scores and surface scores described above respectively. Parameters found to optimize prediction power of surface measures and log-odds ratio scores individually on each of the 5 training sets were used as inputs and the optimal values of *α* found by grid search using the grid: *α = {0.005,0.010…1.0}*. As the numerical values of RSA scores were much lower than the log-odds ratio scores, RSA values were multiplied by 10 to ensure a smooth optimization curve.

### Performance measure

The area under receiver operation curve (AUC) [33] was used as performance measure. An AUC score is the area under the curve obtained by varying the prediction threshold and plotting true positive rate against the false positive rate. The AUC score were calculated per structure bases, to ensure that predicting all residues as either epitope or non-epitope residues results in an AUC score of 0.5. The performance of each homology groups was measured as the average AUC score of the interacting antigen chains in the group, and the overall performance as the average AUC score of the 25 homology-groups as described in [12]. The performances reported are on evaluation sets (not used for training).

### Correlation between log-odds ratio scores and surface scores

The correlation between log-odds ratio scores and surface scores were assessed using the Pearson correlation coefficient (PCC). As for the AUC scores, a PCC score were calculated for each antigen, averaged over each of the 25 homology-groups and the overall correlation was computed as the average PCC of the homology groups.

### Inclusion of multiple epitopes in benchmark

The evaluation procedure for each complex was changed to accommodate multiple epitopes within each homology groups (proteins). Multiple alignments of sequences within each homology groups were made, and a new AUC score for each of the complexes were calculated by excluding non-epitope annotated residues, annotated as epitope in one or more of the other complexes. The new procedure only affected performance of the homology-groups in the data sets that contains multiple epitopes (Table S1 and Table S2).

### Prediction by PEPITO

The PEPITO were implemented based on [18] in python. We compared the prediction values of this script to the output of the BePro server (http://pepito.proteomics.ics.uci.edu/) for several different structures and in all cases a perfect correlation (*r^2^ = 1.00*) was observed.

### Prediction by ElliPro

The 52 proteins in the evaluation dataset were submitted to the ElliPro prediction server (tools.immuneepitope.org/tools/ElliPro/iedb_input) [19]. Clicking the “Click here to view residue scores” button retrieved the residue scores used for performance evaluation.

### Filtering the benchmark dataset for entries used for training

To avoid overestimation of prediction performance for the different prediction tools benchmarked here, antigens in the evaluation dataset were blasted against the individually dataset used for training the methods and antigens with and E-values <0.01 was removed. Refer to supplementary materials Table S4 and Table S5 for details on which antigens were filtered for the individual methods. The SEPPA training set was obtained from: http://lifecenter.sgst.cn/seppa/download.php?id=seppa, the Epitopia data set from http://www.tau.ac.il/~talp/EpitopePrediction, the EPCES from [27] and the EPSVR data from http://sysbio.unl.edu/services/EPSVR/training.tar.gz. PEPITO was developed using the DiscoTope dataset and ElliPro is the webserver implementation of Thornton's method [23], hence these two methods have not been trained on any of the structures in the evaluation dataset.

### Prediction by SEPPA

Antigen structures were submitted to the SEPPA prediction server (http://lifecenter.sgst.cn/seppa/index.php) [26] and the score files were downloaded and used for evaluation.

### Prediction by Epitopia

Antigens were submitted to the Epitopia server: http://epitopia.tau.ac.il/index.html and the output retrieved. The performance was evaluated based on the immuniginicity score, which gave slightly better results compared to the probability score also provided by Epitopia (data not shown).

### Prediction by EPCES and EPSVR

Predictions by EPCES and EPSVR were kindly provided by Chi Zhang, Assistant Professor at School of Biological Sciences, Center for Plant Science Innovation, University of Nebraska.

### Statistical comparison of performance

A one tailed paired t-test, pairing the different homology-groups within a given benchmark data set, was used to compare performances between the different methods.

### Defining unique epitopes

Unique epitopes were found using the method described in [34]. In short: Each epitope-paratope interface was translated to a 400 dimensional “interaction vector”. The vector holds the frequency of the interacting amino acids in the epitope and paretope i.e. the first dimension is assigned the frequency of alanines in the epitope in contact with alanines in the paratope, the second dimension the frequency of alanine–valine contacts and so forth. Epitopes with an angle below 0.8 radians are defined as similar.

## Supporting Information

Figure S1 **Performance of surface corrected log-odds scores.** 3 new sets of log-odds ratios were calculated by excluding residues with a relative surface accessibility (RSA) below 0.01, 0.05 and 0.10 respectively (see text). All parameters were retrained as described in Materials and Methods.
(EPS)

Figure S2 **Influence on weight on surface measure when combined to surface corrected log-odds ratios scores.** The methods were combined with each of the 5 different surface measures and the weight parameter α were optimized to give the best prediction performance of the combined methods as described in Material and Methods. The figure displays the α value average over the 5 training sets for each of the combined methods and illustrates that the α value in general increases concurrently with increase in the RSA threshold for calculating log-odds ratios. An increase of α means that more weight is put on surface measures.
(EPS)

Table S1 **The DiscoTope data set.** The DiscoTope dataset described in [12] was subject to manual annotation, noting number of PDB files, number of unique epitopes, protein name and biological unit for each of the 25 homology-groups. The table gives the features and performance measure of each entry in the DiscoTope dataset. Columns from left to right: 1) entry id in the protein database (PDB). The character after the dot indicates which chain interacts with the antibody. 2) Indicates to which homology group the PDB entry belongs. 3) Training partition of the dataset is used for cross-validation (5 in total, see text). 4) Protein name. Note, that homology group 3 comprises two different protein names. Entries for all other homology groups have the same protein annotation. 5) The in vivo biological unit that the entry is a part of. 6) Notes on content of PDB files available. 7) Number of residues comprising the epitope in the PDB entry. 8) Number of residues available in the PDB file for the antigen chain interacting with the antibody. 9) The AUC performance of the *DiscoTope* method. 10) The performance of the improved DiscoTope-2.0 method [AUC]. 11) The AUC performance of the *DiscoTope-2.0* method evaluated using a new benchmark setup (see text).
(PDF)

Table S2 **Overview of surface exposure measures.** Different surface measures were tested and trained for their ability to discriminate epitope from non-epitope residues (for details see text).
(PDF)

Table S3 **Results of cross-validation of surface exposure measures.** The data were split in 5 datasets, where 4 were used for training of parameters and the remaining dataset for evaluation of surface measure performance. The surface exposure measures were tested for their ability to predict epitopes, and parameters were estimated by a one-dimensional grid search as described in Materials and Methods.
(PDF)

Table S4 **Performance of** ***DiscoTope-1.2*** **,** ***ElliPro*** **,** ***SEPPA*** **,** ***Epitopia*** **,** ***EPCES*** **,** ***EPSVR*** **,** ***PEPITO*** **and** ***DiscoTope-2.0*** **on the evaluation dataset.** The table gives the features and performance measure of each entry in the dataset. Columns from left to right: 1) entry id in the protein database (PDB). The character(s) after the dot indicates which chain(s) interacts with the antibody. 2) Indicates which homology group the PDB entry belongs to. 3) Antigen names. 4) The in vivo biological unit that the entry is a part of. 5) Number of residues comprising the epitope in the PDB entry. 6) Number of residues available in the PDB file for the antibody interacting antigen chain(s). 7) The performance of the DiscoTope-1.2 (original) method [AUC]. 8) Performance of the *ElliPro* prediction server [AUC] 9) Performance of the *SEPPA* prediction method [AUC] 10) Performance of the *Epitopia* prediction server [AUC] 11) Performance of *EPCES* [AUC] 12) Performance of *EPSRV* [AUC] 13) Performance of the *PEPITO* (BePro) prediction server [AUC], 14) The performance of the improved *DiscoTope-2.0* method [AUC] and 15) The performance of the *DiscoTope-2.0* method evaluated using a new benchmark setup (see text) [AUC]. Entries with high sequence similarity to data used for training of the *SEPPA*, *Epitopia*, *EPCES*, and *EPSVR* methods are marked with “used for training”.
(PDF)

Table S5 **Predictive positive value (PPV) and sensitivity for** ***DiscoTope-2.0*** **,** ***SEPPA*** **,** ***Epitopia*** **and** ***EPCES*** **methods calculated for the subset of the benchmark dataset not sharing sequence similarity to the dataset used for training the different methods.**
(DOCX)
