# Supplementary material for: Reliable B Cell Epitope Predictions: Impacts of Method Development and Improved Benchmarking
Source: PLoS Comput Biol. 2012 Dec 27;8(12):e1002829. doi: 10.1371/journal.pcbi.1002829 (PMC3531324; doi:10.1371/journal.pcbi.1002829)
Supplement: Table S1 — The DiscoTope data set. The DiscoTope dataset described in [12] was subject to manual annotation, noting number of PDB files, number of unique epitopes, protein name and biological unit for each of the 25 homology-groups. The table gives the features and performance measure of each entry in the DiscoTope dataset. Columns from left to right: 1) entry id in the protein database (PDB). The character after the dot indicates which chain interacts with the antibody. 2) Indicates to which homology group the PDB entry belongs. 3) Training partition of the dataset is used for cross-validation (5 in total, see text). 4) Protein name. Note, that homology group 3 comprises two different protein names. Entries for all other homology groups have the same protein annotation. 5) The in vivo biological unit that the entry is a part of. 6) Notes on content of PDB files available. 7) Number of residues comprising the epitope in the PDB entry. 8) Number of residues available in the PDB file for the antigen chain interacting with the antibody. 9) The AUC performance of the DiscoTope method. 10) The performance of the improved DiscoTope-2.0 method [AUC]. 11) The AUC performance of the DiscoTope-2.0 method evaluated using a new benchmark setup (see text). (PDF) [file pcbi.1002829.s003.pdf]

Supplementary Table 1. The DiscoTope data set.

PDB id	Hom. group	Training partition	Protein name	Biological unit	Notes	Epitope size	Antigen size	*DiscoTope*	*DiscoTope-2.0*	*Redefined benchmark*
**1A2Y.C**	1	2	Lysozyme	Single chain	Overlapping epitopes. 59 of 129 residues in total are involved in antibody binding	15	129	0.684	0.661	0.841*
**1BQL.Y**	1	2				13	129	0.743	0.776	0.881*
**1BVK.C**	1	2				16	129	0.676	0.696	0.868*
**1C08.C**	1	2				17	129	0.755	0.642	0.822*
**1DQJ.C**	1	2				21	129	0.762	0.662	0.834*
**1DZB.X**	1	2				18	129	0.755	0.731	0.869*
**1FDL.Y**	1	2				14	129	0.663	0.639	0.844*
**1G7H.C**	1	2				16	129	0.691	0.691	0.859*
**1G7I.C**	1	2				15	129	0.663	0.660	0.846*
**1G7J.C**	1	2				15	129	0.662	0.657	0.843*
**1G7L.C**	1	2				15	129	0.668	0.660	0.846*
**1G7M.C**	1	2				15	129	0.694	0.687	0.855*
**1IC4.Y**	1	2				18	129	0.763	0.662	0.840*
**1IC5.Y**	1	2				16	129	0.740	0.632	0.826*
**1IC7.Y**	1	2				17	129	0.763	0.643	0.826*
**1J1O.Y**	1	2				19	129	0.767	0.673	0.843*
**1J1P.Y**	1	2				20	129	0.772	0.670	0.841*
**1J1X.Y**	1	2				19	129	0.760	0.673	0.844*
**1JHL.A**	1	2				11	129	0.821	0.783	0.928*
**1KIP.C**	1	2				15	129	0.674	0.663	0.851*
**1KIQ.C**	1	2				15	129	0.670	0.667	0.853*
**1KIR.C**	1	2				14	129	0.743	0.725	0.887*
**1MEL.L**	1	2				22	127	0.681	0.681	0.827*
**1MLC.E**	1	2				16	129	0.777	0.827	0.899*
**1NBY.C**	1	2				19	129	0.708	0.586	0.767*
**1NBZ.C**	1	2				19	129	0.707	0.610	0.787*
**1NDG.C**	1	2				21	129	0.752	0.665	0.834*
**1NDM.C**	1	2				18	129	0.752	0.637	0.818*
**3HFL.Y**	1	2				14	129	0.814	0.816	0.887*

**PDB id**	**Hom. group**	**Training partition**	**Protein name**	**Biological unit**	**Notes**	**Epitope size**	**Antigen size**	***DiscoTope***	***DiscoTope-2.0***	***Redefined benchmark***
**1JPS.T**	2	1	Extracellular domain of tissue factor	3 domains: Extracellular, transmembrane and intracellular	The part of 1JPS.T constituting the epitope is not represented in 1JRH.I.	21	200	0.600	0.684	-
**1JRH.I**	2	1	Extracellular domain of Interferon gamme receptor (IFNgammaR)	Heterodimer IFNGR1 and IFNGR2		15	95	0.582	0.685	-
**1K4C.C**	3	2	KvAP Potassium Channel	Homotetrameric structure of 4 identical subunits	1ORS.C does not structurally align to 1K4C.C and 1K4D.C (RMS = 5.139)	14	103	0.693	0.732	0.877*[T]
**1K4D.C**	3	2				13	103	0.724	0.749	0.887*[T]
**1ORS.C**	3	2				10	132	0.523	0.684	-
**1XIW.A**	4	1	human CD3-epsilon	CD3-epsilon/delta		17	91	0.870	0.915	0.901[T]
**1LK3.A**	5	2	IL-10 monomer	Homodimer		18	136	0.705	0.699	-
**1FJ1.F**	6	1	Outer surface protein A (OspA)		Lipid protein anchored to the membrane	17	251	0.637	0.664	0.698*
**1OSP.O**	6	1				20	251	0.748	0.840	0.850*
**1H0D.C**	7	3	Angiogenin or Ribonuclease 5			17	122	0.497	0.671	-
**1FE8.A**	8	1	Von Willebrand Factor A3 domain	Multimer of a 2050 residues multidomain monomer		19	186	0.731	0.752	0.786*
**1FNS.A**	8	1				12	196	0.907	0.948	0.961*
**1MHP.A**	8	1				16	184	0.764	0.789	0.826*
**1OAK.A**	8	1				13	196	0.889	0.918	0.938*
**1IQD.C**	9	3	Human Factor VIII C2 Domain (C-terminal)	Multimer of a 2332 residues multidomain monomer		16	156	0.785	0.922	-
**1G9M.G**	10	4	gp120 core	Gp160 – Split into Gp41 and Gp120	In all PDB files: The core region (479 residues) where the N-terminus GLY ALA GLY substitutes the V1/V2 and V3 loops	12	305	0.425	0.406	-
**1G9N.G**	10	4				12	306	0.388	0.338	-
**1GC1.G**	10	4				11	297	0.433	0.341	-
**1RZJ.G**	10	4				11	305	0.429	0.398	-
**1RZK.G**	10	4				12	306	0.386	0.334	-

PDB id	Hom. group	Training partition	Protein name	Biological unit	Notes	Epitope size	Antigen size	*DiscoTope*	*DiscoTope-2.0*	*Redefined benchmark*
**1EZV.E**	11	3	Yeast cytochrome C Iron sulfur	Cytochrome complex		17	185	0.829	0.852	0.928[T]
**1KYO.E**	11	3				15	185	0.818	0.835	0.929[T]
**1BJ1.W**	12	1	Vascular Endothelial Growth factor (VEGF) Receptor binding domain	Homodimer		16	94	0.902	0.937	0.921*[T]
**1CZ8.W**	12	1				16	94	0.901	0.934	0.928*[T]
**1TZH.V**	12	1				13	94	0.969	0.970	0.971*[T]
**1A14.N**	13	3	Neuraminidase	Homotetramer	In all PDB files: Residue 82-468 of 470 residues	17	388	0.842	0.937	0.952*[T]
**1NCA.N**	13	3				21	389	0.848	0.948	0.960*[T]
**1NCB.N**	13	3				20	389	0.833	0.941	0.953*[T]
**1NCC.N**	13	3				21	389	0.882	0.962	0.969*[T]
**1NCD.N**	13	3				19	389	0.853	0.947	0.970*[T]
**1NMC.N**	13	3				15	388	0.844	0.949	0.960*[T]
**2JEL.P**	14	2	Hpr			15	85	0.595	0.670	-
**1OTS.A**	15	3	CLC chloride channels	Homodimer		9	444	0.823	0.837	0.796[T]
**1N8Z.C**	16	2	Extracellular regions of rat HER2	Homodimer	In PDB file Res 22-635 of 1257	17	581	0.593	0.810	-
**1TPX.A**	17	4	Ovine Prion protein (PrP)		In all PDB files: Residue 127-228 of 256 residues	18	102	0.540	0.407	-
**1TQB.A**	17	4				18	102	0.540	0.414	-
**1TQC.A**	17	4				19	102	0.550	0.433	-
**1AR1.B**	18	4	Cytochrome c oxidase type aa3, bacterial	Cytochrome complex (3-5 subunits)	In PDB file: Subunits I and II	15	252	0.551	0.673	0.763[T]
**1OAZ.A**	19	4	Thioredoxin 1	Monomer		16	115	0.554	0.652	-
**1NFD.B**	20	4	T-cell receptor	Heterodimer		12	239	0.914	0.992	0.993[T]
**2HMI.B**	21	5	HI Subunit of HIV-1 Reverse transcriptase	Heterodimer		9	430	0.880	0.909	0.881[T]
**1EGJ.A**	22	5	Cytokine Receptor, common Beta chain precursor	Heterodimer	In PDB file: Domain 4, residue 133-233.	12	101	0.760	0.851	-
**1EO8.A**	23	5	Hemagglutinin	Homotrimer of disulfide linked HA1-HA2		17	319	0.213	0.156	0.170[T]
**1QFU.A**	23	5				20	317	0.319	0.268	0.291[T]
**1FSK.A**	24	5	BET V 1-A (Major pollen allergen Birch)	Monomer		17	159	0.813	0.825	-
**1TY6.A**	25	5	Integrin Alpha-IIB	Heterodimer		19	452	0.862	0.846	-

* Entries affected by multiple epitopes in homology group. See text. Note that a homology group can have multiple entries comprising the same epitope.
T Entries were additional structural information to the antibody interacting chain were available.

Table S1. The DiscoTope data set. The DiscoTope dataset described in [12] was subject to manual annotation, noting number of PDB files, number of unique epitopes, protein name and biological unit for each of the 25 homology-groups. The table gives the features and performance measure of each entry in the DiscoTope dataset. Columns from left to right: 1) entry id in the protein database (PDB). The character after the dot indicates which chain interacts with the antibody. 2) Indicates to which homology group the PDB entry belongs. 3) Training partition of the dataset is used for cross-validation (5 in total, see text). 4) Protein name. Note, that homology group 3 comprises two different protein names. Entries for all other homology groups have the same protein annotation. 5) The in vivo biological unit that the entry is a part of. 6) Notes on content of PDB files available. 7) Number of residues comprising the epitope in the PDB entry. 8) Number of residues available in the PDB file for the antigen chain interacting with the antibody. 9) The performance of the *DiscoTope* method [AUC]. 10) The AUC performance of the improved DiscoTope-2.0 method. 11) The AUC performance of the *DiscoTope-2.0* method evaluated using a new benchmark setup (see text).
