# Supplementary material for: Reliable B Cell Epitope Predictions: Impacts of Method Development and Improved Benchmarking
Source: PLoS Comput Biol. 2012 Dec 27;8(12):e1002829. doi: 10.1371/journal.pcbi.1002829 (PMC3531324; doi:10.1371/journal.pcbi.1002829)
Supplement: Table S2 — Overview of surface exposure measures. Different surface measures were tested and trained for their ability to discriminate epitope from non-epitope residues (for details see text). (PDF) [file pcbi.1002829.s004.pdf]

Method acronym	Description	Scoring function	Parameter
**FS**	Full sphere neighbor count.	$FS(r,k)$	k
**UHS**	Upper half-sphere neighbor count defined as the half-sphere around the residues C-alpha atom containing the side-chain	$UHS(r,k)$	k
**Ta**	Count of neighbors with any atom within T distance from any atom of the query residue	$Ta(r,T)$	T
**RSA**	Relative surface accessibility	$RSA(r) = \frac{SA}{Max(SA)}$ SA = surface accessibility	None
**HSE**	Weighted upper and lower half-sphere as described in [22].	$HSE(r,k) = -0.5 * UHS(r,k) - 0.25 * DHS(r,k)$	k

Table S2. Overview of surface exposure measures. Different surface measures were tested and trained for their ability to discriminate epitope from non-epitope residues (for details see text).
