# Supplementary material for: Reliable B Cell Epitope Predictions: Impacts of Method Development and Improved Benchmarking
Source: PLoS Comput Biol. 2012 Dec 27;8(12):e1002829. doi: 10.1371/journal.pcbi.1002829 (PMC3531324; doi:10.1371/journal.pcbi.1002829)
Supplement: Table S3 — Results of cross-validation of surface exposure measures. The data were split in 5 datasets, where 4 were used for training of parameters and the remaining dataset for evaluation of surface measure performance. The surface exposure measures were tested for their ability to predict epitopes, and parameters were estimated by a one-dimensional grid search as described in Materials and Methods. (PDF) [file pcbi.1002829.s005.pdf]

Training set	Surface measure	parameter	AUC on evaluation set
**2,3,4,5**	FS	*k* = 18	0.6699
	UHS	*k* = 14	0.6092
	Ta	*T* = 18	0.6783
	RSA		0.6437
	HSE	*k* = 18	0.6576
**1,3,4,5**	FS	*k* = 18	0.6875
	UHS	*k* = 14	0.6459
	Ta	*T* = 16	0.6935
	RSA		0.664
	HSE	*k* = 16	0.6719
**1,2,4,5**	FS	*k* = 18	0.7047
	UHS	*k* = 14	0.6992
	Ta	*T* = 16	0.7071
	RSA		0.6827
	HSE	*k* = 18	0.7035
**1,2,3,5**	FS	*k* = 22	0.6230
	UHS	*k* = 14	0.6343
	Ta	*T* = 16	0.6292
	RSA		0.6347
	HSE	*k* = 18	0.6481
**1,2,3,4**	FS	*k* = 22	0.7051
	UHS	*k* = 12	0.7041
	Ta	*T* = 18	0.7234
	RSA		0.6962
	HSE	*k* = 18	0.7402

Table S3. Results of cross-validation of surface exposure measures. The data were split in 5 datasets, where 4 were used for training of parameters and the remaining dataset for evaluation of surface measure performance. The surface exposure measures were tested for their ability to predict epitopes, and parameters were estimated by a one-dimensional grid search as described in methods.
