# Supplementary material for: Reliable B Cell Epitope Predictions: Impacts of Method Development and Improved Benchmarking
Source: PLoS Comput Biol. 2012 Dec 27;8(12):e1002829. doi: 10.1371/journal.pcbi.1002829 (PMC3531324; doi:10.1371/journal.pcbi.1002829)
Supplement: Table S4 — Performance of DiscoTope-1.2 , ElliPro , SEPPA , Epitopia , EPCES , EPSVR , PEPITO and DiscoTope-2.0 on the evaluation dataset. The table gives the features and performance measure of each entry in the dataset. Columns from left to right: 1) entry id in the protein database (PDB). The character(s) after the dot indicates which chain(s) interacts with the antibody. 2) Indicates which homology group the PDB entry belongs to. 3) Antigen names. 4) The in vivo biological unit that the entry is a part of. 5) Number of residues comprising the epitope in the PDB entry. 6) Number of residues available in the PDB file for the antibody interacting antigen chain(s). 7) The performance of the DiscoTope-1.2 (original) method [AUC]. 8) Performance of the ElliPro prediction server [AUC] 9) Performance of the SEPPA prediction method [AUC] 10) Performance of the Epitopia prediction server [AUC] 11) Performance of EPCES [AUC] 12) Performance of EPSRV [AUC] 13) Performance of the PEPITO (BePro) prediction server [AUC], 14) The performance of the improved DiscoTope-2.0 method [AUC] and 15) The performance of the DiscoTope-2.0 method evaluated using a new benchmark setup (see text) [AUC]. Entries with high sequence similarity to data used for training of the SEPPA, Epitopia, EPCES, and EPSVR methods are marked with “used for training”. (PDF) [file pcbi.1002829.s006.pdf]

PDB id	Hom. Group	Protein Name	Biological Unit	Epitope Size	Antigen size	*DiscoTope-1.2*	*ElliPro*	*SEPPA*	*Epitopia*	*EPCES*	*EPSVR*	*PEPITO*	*DiscoTope-2.0*	*Redefined benchmark*
1YNT.F	1	Major Surface Antigen p30		19	252	0.491	0.710	0.496	0.577	0.737	*Used for training*	0.507	0.410	*NA*
2NYY.A	2	Botulinum Neurotoxin Type A		23	1268	0.617	0.769	0.852	0.833	0.700	*Used for training*	0.735	0.795	*NA*
2NZ9.A	2			25	1268	0.597	0.752	0.706	0.796	0.716	*Used for training*	0.728	0.789	*NA*
3I50.E	3	Envelope Glycoprotein		12	273	0.577	0.945	0.983	0.473	0.385	*Used for training*	0.834	0.962	*NA*
1Z3G.A	4	Ookinete Surface Protein PVS25		19	173	0.689	0.759	0.751	0.744	0.787	*Used for training*	0.761	0.813	*NA*
3RHW.A ,B,C,D,E	5	Avermectin-Sensitive Glutamate-Gated Chloride Channel GLUCL	chain A,B,C,D,E homopentamer	19	340	0.661	0.714	0.732	0.787	*Failed*	*Failed*	0.702	0.669	*NA*
3RI5.A,B ,C,D,E	5			21	339	0.659	0.720	0.704	0.783	*Failed*	*Failed*	0.7fir02	0.671	*NA*
3RIA.A, B,C,D,E	5			21	340	0.648	0.707	0.723	0.781	*Failed*	*Failed*	0.691	0.659	*NA*
3RIF.A,B ,C,D,E	5			19	341	0.667	0.722	0.719	0.793	*Failed*	*Failed*	0.700	0.672	*NA*
1AFV.A	6	Human Immunodeficiency Virus Type 1 Capsid		14	151	0.526	0.540	*Used for training*	*Used for training*	*Used for training*	0.393	0.626	0.713	*NA*
3MJ9.A	7	Junctional Adhesion Molecule-Like		24	229	0.805	0.560	*Used for training*	*Used for training*	*Used for training*	*Used for training*	0.785	0.803	*NA*
1BGX.T	8	Taq Dna Polymerase		52	828	0.739	0.495	*Used for training*	*Used for training*	*Used for training*	*Used for training*	0.738	0.693	*NA*
2R56.A, B	9	Beta-Lactoglobulin	chain A,B homodimer	21	159	0.583	0.666	0.682	0.659	0.629	*Used for training*	0.645	0.661	0.651*
3BSZ.E,F	9	Plasma Retinol-Binding Protein	chain, A,B,C,D, homo-4-mer in complex with chain F	11	174	0.658	0.705	0.639	0.587	0.498	*Used for training*	0.633	0.628	0.672*T
2I9L.I	10	Virion Membrane Protein M25		14	173	0.535	0.785	0.730	0.661	0.445	0.187	0.652	0.770	*NA*
1RVF.1, 2,3	11	Human Rhinovirus 14 Coat Protein	chain 1,2,3 hetorotrimer	12	273	0.810	0.679	0.677	*Used for training*	0.821	0.733	0.838	0.865	*NA*
3G04.C	12	Thyrotropin Receptor		23	228	0.416	0.411	0.442	0.507	0.750	0.813	0.445	0.438	0.466*

PDB id	Hom. Group	Protein Name	Biological Unit	Epitope Size	Antigen size	*DiscoTope-1.2*	*ElliPro*	*SEPPA*	*Epitopia*	*EPCES*	*EPSVR*	*PEPITO*	*DiscoTope-2.0*	*Redefined benchmark*
3KJ4.A	12	Reticulon-4 Receptor		19	283	0.514	0.722	0.735	0.661	0.544	0.438	0.467	0.366	0.343*
3O0R.B, C	13	Nitric Oxide Reductase Subunit B	C,B hetorodimer	9	449	0.885	0.868	0.880	0.834	0.723	0.380	0.900	0.891	*NA*
2Q8A.A	14	Apical Membrane Antigen 1		21	316	0.811	0.776	*Used for training*	0.673	0.761	*Used for training*	0.790	0.779	*NA*
2Q8B.A	14			21	292	0.886	0.822	*Used for training*	0.630	0.749	*Used for training*	0.857	0.867	*NA*
3GI8.C	15	Uncharacterized Protein Mj0609		16	435	0.920	0.761	0.871	0.899	0.645	0.326	0.912	0.945	*NA*
3GI9.C	15			16	437	0.921	0.780	0.906	0.918	0.569	0.331	0.919	0.949	*NA*
3NCY.C, D	15	Adic	chain C,D homodimer	12	420	0.928	0.664	0.943	0.960	0.920	0.843	0.907	0.934	*NA*
2DD8.S	16	Spike Glycoprotein		19	192	0.634	0.629	*Used for training*	0.682	0.730	*Used for training*	0.679	0.720	0.759*
2GHW.A	16			24	191	0.729	0.755	*Used for training*	0.734	0.750	*Used for training*	0.836	0.901	0.896*
3BGF.S	16			15	179	0.611	0.636	*Used for training*	0.616	0.763	*Used for training*	0.649	0.666	0.724*
2XQY.A	17	Envelope Glycoprotein H		19	464	0.691	0.541	0.701	0.605	0.710	0.503	0.674	0.667	*NA*
2NR6.A	18	Aspartic Protease BLA G 2		18	330	0.725	0.783	*Used for training*	0.877	0.869	*Used for training*	0.710	0.695	0.706*
3LIZ.A	18			18	329	0.647	0.809	*Used for training*	0.538	0.499	*Used for training*	0.693	0.712	0.726*
2ZJS.Y	19	Preprotein Translocase Secy Subunit	chain Y,E hetorodimer	11	415	0.912	0.946	0.929	0.946	0.732	0.521	0.938	0.959	0.945[T]
2UZI.R	20	Gtpase Hras		18	166	0.724	0.685	*Used for training*	0.760	0.643	*Used for training*	0.701	0.641	*NA*
2VH5.R	20			17	166	0.743	0.696	*Used for training*	0.835	0.644	*Used for training*	0.717	0.668	*NA*
2XTJ.A	21	Proprotein Convertase Subtilisin/Kexin Type 9	chain A,P hetorodimer	17	243	0.749	0.812	0.569	*Used for training*	0.929	0.686	0.820	0.903	0.927*[T]
3H42.B, A	21		chain A,B hetorodimer	22	492	0.719	0.586	0.635	*Used for training*	0.806	0.777	0.716	0.725	0.734*
3R1G.B	22	Beta-Secretase 1		19	381	0.721	0.913	0.797	0.634	0.768	0.713	0.754	0.726	*NA*
2ZCH.P	23	Prostate-Specific Antigen		16	237	0.837	0.674	*Used for training*	0.459	0.698	*Used for training*	0.794	0.758	0.776*
2ZCK.P	23			20	237	0.822	0.677	*Used for training*	0.473	0.690	*Used for training*	0.800	0.748	0.763*

PDB id	Hom. Group	Protein Name	Biological Unit	Epitope Size	Antigen size	*DiscoTope-1.2*	*ElliPro*	*SEPPA*	*Epitopia*	*EPCES*	*EPSVR*	*PEPITO*	*DiscoTope-2.0*	*Redefined benchmark*
2ZCL.P	23			18	237	0.833	0.671	*Used for training*	0.451	0.696	*Used for training*	0.801	0.765	0.780*
3BN9.B	23	Membrane-Type Serine Protease 1		24	241	0.614	0.585	*Used for training*	0.641	0.742	*Used for training*	0.599	0.547	0.555*
3GRW.A	24	Fibroblast Growth Factor Receptor 3		35	206	0.539	0.340	0.478	*Used for training*	0.541	*Used for training*	0.547	0.559	*NA*
3L95.Y	25	Neurogenic Locus Notch Homolog Protein 1		22	218	0.524	0.568	0.394	0.445	0.544	0.367	0.501	0.443	*NA*
3MXW.A	26	Sonic Hedgehog Protein		22	155	0.649	0.524	0.823	0.484	0.635	0.609	0.642	0.639	*NA*
2R4R.A	27	Beta-2 Adrenergic Receptor		11	216	0.964	0.885	0.969	0.906	0.973	0.926	0.975	0.978	*NA*
2R4S.A	27			12	216	0.967	0.890	0.970	0.895	0.977	0.937	0.978	0.981	*NA*
3KJ6.A	27			11	222	0.971	0.900	0.967	0.922	0.962	0.972	0.976	0.979	*NA*
2OZ4.A	28	Intercellular Adhesion Molecule 1		18	265	0.790	0.401	0.573	0.275	0.746	0.692	0.786	0.789	*NA*
2FD6.U	29	Urokinase Plasminogen Activator Surface Receptor	chain U,A hetorodimer	13	249	0.752	0.682	*Used for training*	0.598	0.745	*Used for training*	0.790	0.706	0.752[T]
3G6J.B,A	30	Complement C3 Alpha Chain	dimers of A,B hetorodimer, (A,B) (C,D)	12	903	0.846	0.669	0.851	*Used for training*	0.850	0.828	0.892	0.917	*NA*
3FMG.A, A,A	31	Glycoprotein Vp7	chain A,A,A homotrimer	7	237	0.642	0.693	0.587	*Used for training*	0.868	0.414	0.675	0.581	*NA*
3PGF.A	32	Maltose-Binding Periplasmic Protein		24	358	0.506	0.367	0.486	0.560	0.539	0.673	0.439	0.337	*NA*
2J88.A	33	Hyaluronongluco saminidase		10	319	0.961	0.992	*Used for training*	0.487	0.579	0.761	0.989	0.989	*NA*

* Entries affected by multiple epitopes in homology group. See text. Note that a homology group can have multiple entries comprising the same epitope.

[T] Entries were additional structural information to the antibody interacting chain(s) were available.

Supplementary Table S4. Performance of *DiscoTope-1.2*, *ElliPro*, *SEPPA*, *Epitopia, EPCES, EPSVR, PEPITO* and *DiscoTope-2.0* on the evaluation dataset. The table gives the features and performance measure of each entry in the dataset. Columns from left to right: 1) entry id in the protein database (PDB). The character(s) after the dot indicates which chain(s) interacts with the antibody. 2) Indicates which homology group the PDB entry belongs to. 3) Antigen names. 4) The in vivo biological unit that the entry is a part of. 5) Number of residues comprising the epitope in the PDB entry. 6) Number of residues available in the PDB file for the antibody interacting antigen chain(s). 7) The performance of the DiscoTope-1.2 (original) method [AUC]. 8) Performance of

the ElliPro prediction server [AUC] 9) Performance of the SEPPA prediction method [AUC] 10) Performance of the Epitopia prediction server [AUC] 11) Performance of EPCES [AUC] 12) Performance of EPSRV [AUC] 13) Performance of the PEPITO (BePro) prediction server [AUC], 14) The performance of the improved DiscoTope-2.0 method [AUC] and 15) The performance of the DiscoTope-2.0 method evaluated using a new benchmark setup (see text) [AUC]. Entries with high sequence similarity to data used for training of the *SEPPA, Epitopia, EPCES,* and *EPSVR* methods are marked with “used for training”.
