# Supplementary material for: Reliable B Cell Epitope Predictions: Impacts of Method Development and Improved Benchmarking
Source: PLoS Comput Biol. 2012 Dec 27;8(12):e1002829. doi: 10.1371/journal.pcbi.1002829 (PMC3531324; doi:10.1371/journal.pcbi.1002829)
Supplement: Table S5 — Predictive positive value (PPV) and sensitivity for DiscoTope-2.0 , SEPPA , Epitopia and EPCES methods calculated for the subset of the benchmark dataset not sharing sequence similarity to the dataset used for training the different methods. (DOCX) [file pcbi.1002829.s007.docx]

Table S5. Predictive positive value (PPV) and sensitivity for *DiscoTope-2.0*, *SEPPA*, *Epitopia* and *EPCES* methods calculated for the subset of the benchmark dataset not sharing sequence similarity to the dataset used for training the different methods.

| **Dataset**  **N_struct_/N_groups_** | **Method** | **#Top 15 Residues** | | **# Top 30 Residues** | |
| --- | --- | --- | --- | --- | --- |
|  |  | **PPV** | **Sens** | **PPV** | **Sens** |
| 34/23 | *DiscoTope-2.0* | 0.182 | 0.168 | 0.145 | 0.262 |
|  | SEPPA | 0.142 | 0.135 | 0.140 | 0.258 |
| 43/25 | *DiscoTope-2.0* | 0.197 | 0.195 | 0.161 | 0.306 |
|  | Epitopia | 0.093 | 0.071 | 0.101 | 0.164 |
| 49/29 | *DiscoTope-2.0* | 0.189 | 0.188 | 0.155 | 0.296 |
|  | EPCES | 0.163 | 0.125 | 0.147 | 0.221 |
| 24/18 | *DiscoTope-2.0* | 0.189 | 0.205 | 0.144 | 0.303 |
|  | EPSVR | 0.075 | 0.068 | 0.057 | 0.101 |

# Residues gives the number of highest scoring prediction included for each antigen, PPV gives the predictive positive value (true positives)/(predicted positives)), and Sens gives the sensitivity (true positives)/(actual positives)).
